# Competitive Drivers of Atrial Fibrillation: The Interplay Between Focal Drivers and Multiwavelet Reentry

**DOI:** 10.3389/fphys.2021.633643

**Published:** 2021-03-16

**Authors:** Richard T. Carrick, Bryce E. Benson, Oliver R. J. Bates, Peter S. Spector

**Affiliations:** ^1^College of Medicine, University of Vermont, Burlington, VT, United States; ^2^College of Engineering and Mathematical Sciences, University of Vermont, Burlington, VT, United States; ^3^College of Engineering, Boston University, Boston, MA, United States

**Keywords:** atrial fibrillation, fibrillatory conduction, multi-wavelet reentry, rotors, focal driver, computational model

## Abstract

**Background:**

There is debate whether human atrial fibrillation is driven by focal drivers or multiwavelet reentry. We propose that the changing activation sequences surrounding a focal driver can at times self-sustain in the absence of that driver. Further, the relationship between focal drivers and surrounding chaotic activation is bidirectional; focal drivers can generate chaotic activation, which may affect the dynamics of focal drivers.

**Methods and Results:**

In a propagation model, we generated tissues that support structural micro-reentry and moving functional reentrant circuits. We qualitatively assessed (1) the tissue’s ability to support self-sustaining fibrillation after elimination of the focal driver, (2) the impact that structural-reentrant substrate has on the duration of fibrillation, the impact that micro-reentrant (3) frequency, (4) excitable gap, and (5) exposure to surrounding fibrillation have on micro-reentry in the setting of chaotic activation, and finally the likelihood fibrillation will end in structural reentry based on (6) the distance between and (7) the relative lengths of an ablated tissue’s inner and outer boundaries. We found (1) focal drivers produced chaotic activation when waves encountered heterogeneous refractoriness; chaotic activation could then repeatedly initiate and terminate micro-reentry. Perpetuation of fibrillation following elimination of micro-reentry was predicted by tissue properties. (2) Duration of fibrillation was increased by the presence of a structural micro-reentrant substrate only when surrounding tissue had a low propensity to support self-sustaining chaotic activation. Likelihood of micro-reentry around the structural reentrant substrate increased as (3) the frequency of structural reentry increased relative to the frequency of fibrillation in the surrounding tissue, (4) the excitable gap of micro-reentry increased, and (5) the exposure of the structural circuit to the surrounding tissue decreased. Likelihood of organized tachycardia following termination of fibrillation increased with (6) decreasing distance and (7) disparity of size between focal obstacle and external boundary.

**Conclusion:**

Focal drivers such as structural micro-reentry and the chaotic activation they produce are continuously interacting with one another. In order to accurately describe cardiac tissue’s propensity to support fibrillation, the relative characteristics of both stationary and moving drivers must be taken into account.

## Introduction

When the cardiac arrhythmia atrial fibrillation was first identified more than 100 years ago (McMichael, 1982; Silverman, 1994; Flegel, 1995), it was defined in descriptive terms as an atrial rhythm with a perpetually changing pattern of activation (Lip and Beevers, 1995; Nattel et al., 2002). The treatment of atrial fibrillation requires more than the knowledge that activation sequence is changing; it requires knowledge of the mechanism(s) responsible for the arrhythmia perpetuation. Unfortunately, identifying what maintains fibrillation in humans has been challenging. As a result, the mechanism(s) responsible for perpetuation of atrial fibrillation remain hotly debated to this day (Garrey, 1924; Allessie and de Groot, 2014; Narayan and Jalife, 2014). Largely, opinions have been divided between two apparently competing hypotheses: focal drivers (FD) and multiwavelet reentry (MWR). In the FD hypothesis, a stationary driver (e.g., focal rotor) generates waves that propagate to the surrounding tissue in a non-uniform fashion (Scherf et al., 1948; Allessie et al., 1977; Fareh et al., 1998; Ogawa et al., 2002; Oliveira et al., 2007; Narayan et al., 2012). This changing activation confers the irregularity that is emblematic of fibrillation. It is generally tacitly assumed that this, “fibrillatory conduction” (FC), is a purely passive phenomenon; i.e., FC would cease in the absence of a focal driver. In contrast, the MWR hypothesis refers to self-sustaining, moving functional reentrant circuits that do not require the presence of a focal driver (Moe et al., 1964; Kirchhof et al., 1993). The gross observation of changing atrial activation is inadequate to distinguish passively driven FC from active self-sustaining MWR.

We postulated that what is often described as fibrillatory conduction could under some circumstances represent moving functional reentry and as such could sustain in the absence of a focal driver. Furthermore, if MWR and FD can coexist it is possible that the chaotic waves surrounding a focal driver might interact with that driver and have implications for the duration of episodes of fibrillation.

In the present study, we examine structural micro-reentry as a focal driver of fibrillation and use a computational model of propagation to qualitatively study the ability of chaotic activation to sustain after driver elimination, the interactions between these chaotically propagating waves and the spatially fixed structural reentrant substrate, and the impact that these interactions have on the overall duration of fibrillation. We also examined the likelihood that fibrillation will organize into atrial trachycardia after focal ablation.

## Materials and Methods

### Computational Model

In the following experiments, we made use of a previously described computational model of electrical propagation in cardiac tissue (Spector et al., 2011, 2012). Our model represents a simplistic version of cardiac propagation with low computational burden, and is not designed to reproduce the exact details of action potential morphology or tissue architecture. However, despite the simplicity of this model, it is able to reproduce complex emergent behaviors including structural and functional reentry, chaotic propagation, and their interactions. This model combines a diffusion equation for electrotonic current spread with a rule based cellular automaton of cardiomyocyte excitation. Briefly, cells (each representing approximately 1 mm^2^ of cardiac tissue) undergo action potentials when they receive current sufficient to perturb their potential from rest (V_rest_) to above a defined threshold (V_thresh_). Action potentials cause the cell voltage to increase toward peak potential (V_peak_) after which it gradually returns to V_rest_ over a period of time, the action potential duration (APD). While the voltage is above V_thresh_ the cell is refractory to new stimuli. Cells transmit current to their adjacent neighbors, increasing the voltage of each neighbor with a time constant equal to the product of the cell-cell ohmic resistance (R) and the electric charge capacitance of the neighboring cell (C). This design results in appropriate source-sink behavior in which curved waves propagate more slowly than planar waves, and beyond a critical curvature propagation fails due to source-sink mismatch, creating rotation around an unexcited core.

**FIGURE 1:**
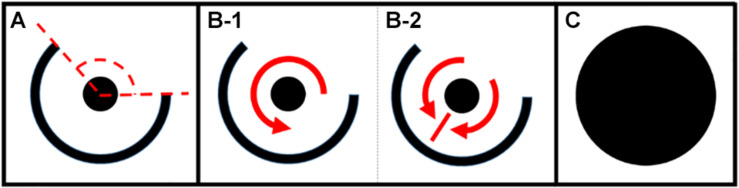
**(A)** Micro-reentrant circuit structure. The center scar (circle) creates a structure around which reentry can occur. The outer ring is a semi-circular scar providing partial protection of the circuit. The dashed lines indicate the angle of exposure (area through which waves can propagate into or out of the circuit). **(B,C)** Anatomic structures for analysis of fibrillation duration. **(B)** Partially protected structural circuit capable of **(B-1)** reentry, or **(B-2)** passive activation of the circuit. **(C)** Ablated micro-reentrant circuit. Fibrillation duration was also assessed in the complete absence of micro-reentrant substrate (not shown) (*the size of the circuit relative to the surrounding tissue is much smaller than drawn here). *This figure relates to experiments 1–5.

The number and arrangement of the cells, as well as the parameter values for each cell, can be adjusted so as to allow simulated cardiac tissue to support various patterns of electrical excitation. In order to test the relationship between structural reentry and multi-wavelet reentry, we simulated rectangular cardiomyocyte monolayers with centralized regions of unexcitable scar tissue. The RC time constant was set at 10 ms for all experiments, with APD varied as described in the series of experiments below. The patterns of scar tissue were specifically designed to support structural micro-reentry (Figures 1A,B). Here, a small circular scar with variable radius (r_scar_) was surrounded by a 1 mm thick protective partial annulus with larger radius (r_barrier_) such that a tract of excitable cells between the two provided the substrate for a reentrant circuit, and an open portion of the protective annulus [defined by the angle of exposure (θ_exposure_)] exposed the circuit to the surrounding cardiac tissue. Micro-reentry could be initiated by appropriately timed programmed stimulation to produce unidirectional block adjacent to the central scar. Structural micro-reentry could then be terminated by focal ablation of the excitable tract between r_scar_ and r_barrier_ (Figure 1C). Model simulations were run on the Vermont Advance Computing Core^1^.

### The Impact of Structural Reentry on Activation in the Surrounding Tissue

To test whether chaotic activation could perpetuate after elimination of focal drivers (experiment 1, Table 1), we simulated 80 × 80 mm cardiomyocyte monolayers with micro-reentrant substrate (r_scar_ = 2 mm, r_barrier_ = 7 mm, θ_exposure_ = 60°). The mean APD of individual monolayers was varied between 65 and 200 ms in 5 ms increments, each with ±10 ms of homogeneously distributed random variation. These parameter ranges were purposely selected to produce episodes of chaotic activation lasting between 0 and 10,000 s in the simulated tissues. In each monolayer, we initiated 500 unique episodes of structural reentry around the central obstacle. One second after initiation, structural reentry was eliminated by ablation. We then measured the mean duration of residual fibrillation in the surrounding monolayer (measured up to a maximum of 10,000 s per episode). These values were compared to predictions made using the fibrillogenicity index (Eq. 1), a previously described metric of cardiac tissue’s propensity to support multi-wavelet reentry (Carrick et al., 2015).

**TABLE 1:** Descriptions of the seven different qualitative experiments that were performed.

Experiment number	1	2	3	4	5	6	7
Constant parameters	θ_exposure_ = 60° r_scar_ = 2 mm APD_structural reentry_ = 60 ms	r_scar_ = 2 mm APD_structural reentry_ = 60 ms	APD_structural reentry_ = 60 ms θ_exposure_ = 60°	r_scar_ = 2 mm θ_exposure_ = 60° APD_surrounding_ = 90 ms	r_scar_ = 2 mm APD_surrounding_ = 90 ms APD_structural reentry_ = 60 ms	APD = 70 ms Inner boundary length = 80 mm	APD = 70 ms Distance between inner and outer boundary = 10 mm
Varied parameters and respective ranges	APD_surround_ = [65 ms, 200 ms, 5 ms]	APD_surround_ = [70 ms, 170 ms, 10 ms], Micro-reentrant substrate with θ_exposure_ = 60°, no micro-reentrant substrate, ablated micro-reentrant substrate	r_scar_ = [0.5 mm, 4.5 mm, 0.5 mm] APD_surrounding_ [50 ms, 160 ms, 10 ms]	APD_structural reentry_ = [40 ms, 100 ms, 10 ms]	θ_exposure_ = [0°, 360°, 15°]	Distance between inner and outer boundary = [10 mm, 80 mm, 5 mm]	Inner boundary radius = [1 mm, 20 mm, 1 mm]
Chaotic propagation initiation method	Micro-reentry	Burst pacing	Burst pacing	Burst pacing	Burst pacing	Burst pacing	Burst pacing
Measured outcome	Duration chaotic propagation	Duration chaotic propagation	% time w/micro-reentry	% time w/micro-reentry	% time w/micro-reentry	Proportion micro-reentry vs. quiescence	Proportion micro-reentry vs. quiescence

*Varied parameter ranges are presented as (lower parameter value, upper parameter value, increment by which the parameter was varied over the range).*

(1) $$F\,b=\frac{A\,r\,e\,a}{B\,o\,u\,n\,d\,a\,r\,y\,L\,e\,n\,g\,t\,h*A\,P\,D}$$

To test whether the presence of structural micro-reentrant substrate impacted duration of MWR (experiment 2, Table 1), we initiated MWR in the above series of monolayers by high frequency burst pacing (100 Hz) from a virtual electrode positioned far from the central scar. The mean APD of individual monolayers was varied between 70 and 170 ms in 10 ms increments, each with ±10 ms of homogeneously distributed random variation. These APD values were chosen to produce chaotic propagation with frequency that was both above and below the frequency of the structural micro-reentry. The central scar pattern was modified in one of three ways: partially protected micro-reentrant substrate (Figure 1A), no micro-reentrant substrate, and focally ablated micro-reentrant substrate (Figure 1C). In each monolayer/scar pattern set-up, we measured the mean duration of 500 unique episodes of MWR measured up to a maximum of 10,000 s per episode.

### The Impact of Multi-Wavelet Reentry on Structural Mirco-Reentry

**FIGURE 2:**
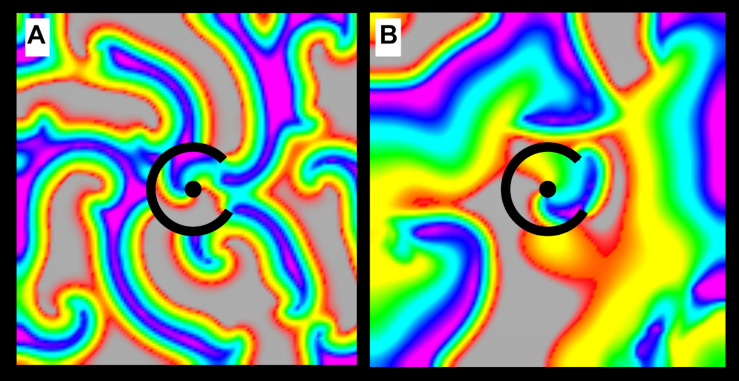
Chaotic activation surrounding a micro-reentrant driver. **(A)** When the APD of the tissue surrounding the micro-reentrant driver is short, wave length is reduced and the frequency of activation in the surrounding tissue is high. **(B)** When the APD in the surrounding tissue is long, wave length is increased and the frequency of activation is low. The interactions between the focal driver and the surrounding activation are influenced by their relative rates [colors represent membrane voltage (purple—maximum voltage, red—threshold voltage, gray—sub-threshold voltage). *This figure relates to experiments 3.

In order to assess the dynamics of structural reentry in coexistence with MWR, we simulated 80 × 80 mm cell monolayers with micro-reentrant substrate (r_barrier_ = 7 mm, r_scar_ = 2 mm, θ_exposure_ = 60°). In these monolayers, the APDs of the central region (*r* ≤ 7 mm, APD_structural reentry_ = 60 ms) and the surrounding monolayer (*r* > 7 mm, APD_surround_ = 90 ms) were varied independently (both with ±10 ms of homogeneously distributed random variation). We tested the likelihood of structural micro-reentry in three separate experiments. In the first (experiment 3, Table 1), micro-reentrant frequency around the central scar was adjusted by varying r_scar_ between 1 and 4.5 mm in increments of 0.5 mm. The frequency of fibrillation in the surrounding monolayer was also adjusted by varying APD_surround_ between 50 and 160 ms in increments of 10 ms. These APD values were chosen to produce chaotic propagation with frequency that was above (Figure 2A), equal to, and below (Figure 2B) the frequency of the structural micro-reentry. In the second (experiment 4, Table 1), the excitable gap of structural micro-reentry was adjusted by varying the APD_structural reentry_ between 40 and 100 ms in 10 ms increments. In the third (experiment 5, Table 1), the percentage of the micro-reentrant substrate surface exposed to fibrillation in the surrounding tissue was adjusted by varying θ_exposure_ between 0° and 360° in 15° increments.

In each of the above experimental monolayer set-ups, we created 50 episodes of MWR by high frequency burst pacing (100 Hz) for 10 s from a virtual electrode positioned far from the central scar. The mean proportion of time in which structural micro-reentry and MWR coexisted was measured for the 10 s period of MWR. Structural reentry was declared present when all cells directly adjacent to the central obstacle were found to be simultaneously undergoing periodic excitation, as defined by Eq. 2. In this case, a threshold for periodic behavior (t_thresh_) of 5 ms was used.

(2) $$t_{\text{thresh}}\geq \left(A\,P\,D_{1}+A\,P\,D_{3}\right)-2\,A\,P\,D_{2}$$

### The Probability of Structural Macro-Reentry After Termination of Multiwavelet Reentry

**FIGURE 3:**
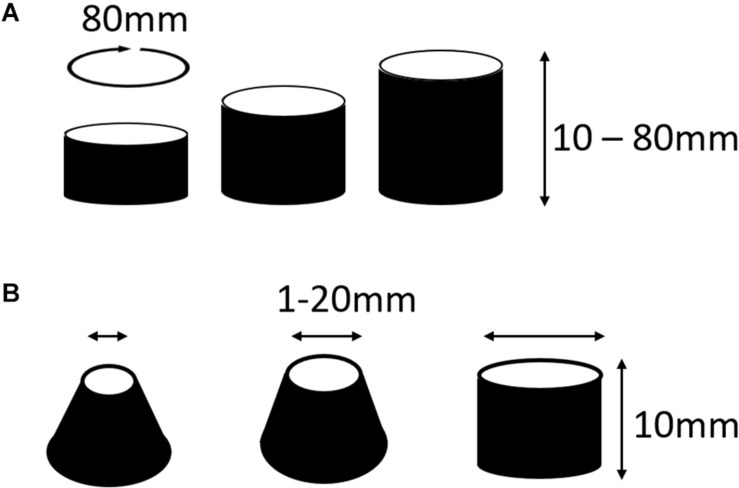
Cylindrical monolayers used to test the likelihood that MWR would terminate in macro-reentrant tachycardia (experiment 6). **(A)** In the first of these experiments the circumference of the cylinders were all equal to 80 mm, the height was varied between 10 and 80 mm in increments of 5 mm. This allowed testing of the relationship of the distance between boundaries and termination of fibrillation into macro-reentrant tachycardia. **(B)** In the second of these experiments there was an annuli of tissue that was consistently 10 mm wide, the radius of the lower boundary was fixed at 20 mm while the upper boundary radius varied from 1 to 20 mm in 1 mm increments. *This figure relates to experiments 6 and 7.

In two separate experiments, we tested the likelihood that MWR would end in macro-reentrant tachycardia (reentry around the ablated central region) as a function of both the distance between, and the relative sizes, of the central scar (e.g., Figure 1A) and the unexcitable external boundary. In the first (experiment 6, Table 1), we simulated cyclindrical monolayers with circumference of 80 mm and height ranging between 10 and 80 mm in increments of 5 mm (Figure 3A). In the second (experiment 7, Table 1), we created an annuli of excitable cardiac tissue with constant width of 10 mm but variable internal radius (between 1 and 20 mm in increments of 1 mm) (Figure 3B). In both experiments, monolayers had APD of 70 ms with ±10 ms of homogeneously distributed random variation. In each monolayer, we initiated 500 unique episodes of MWR by high frequency burst pacing (100 Hz) for 1 s from a randomly positioned virtual electrode. Periodic behavior was declared when all cells were found to be simultaneously undergoing periodic excitation (Eq. 2), and the proportion of episodes in each monolayer that ended in periodic behavior vs. quiescence was recorded.

## Results

### The Impact of a Structural Reentrant Circuit on Fibrillation

**FIGURE 4:**
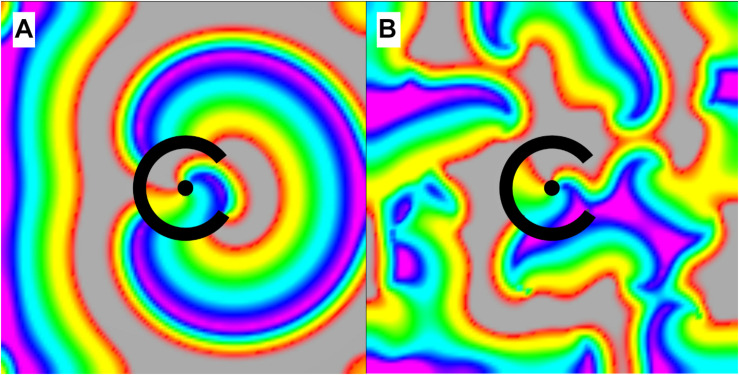
Activation of the tissue surrounding the micro-reentrant driver. **(A)** When the APD of the surrounding tissue was between 90 and 110 ms waves exiting the micro-reentrant driver excited the surrounding tissue (2:1) in an organized fashion, **(B)** for APD less than 90 or greater than 110 ms propagation was chaotic. *When activation was organized, there was no wave break or rotation and hence no circuits in the surrounding tissue to sustain activation following micro-reentrant driver elimination [colors represent membrane voltage (purple—maximum voltage, red—threshold voltage, gray—sub-threshold voltage]. *This figure relates to experiment 1.

**FIGURE 5:**
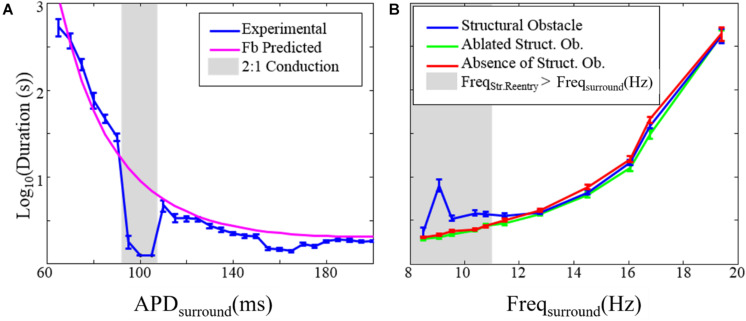
The effect of structural reentry on the duration of fibrillation. **(A)** Duration of fibrillation after elimination of the substrate for structural reentry vs. the APD of the tissue surrounding the structural circuit (APD_surround_). The gray box indicates APD_surround_ values over which 2:1 conduction occurred so that chaotic activation was absent. **(B)** Duration of fibrillation in the presence (blue), absence (red), and after ablation of (green) the substrate for structural reentry. The gray box indicates range over which the frequency of structural reentry was faster than the frequency of fibrillation in the surrounding tissue. Error bars show 95% confidence intervals. *This figure relates to experiments 1 and 2.

In our first simulation of structural micro-reentry (experiment 1, Table 1), the frequency of micro-reentry around the central scar was 10.9 Hz. Structural micro-reentry produced either organized (i.e., tachycardia, Figure 4A) or chaotic propagation (i.e., fibrillation, Figure 4B) depending upon the refractory period of the surrounding cardiac tissue. When the APD in the cells of the surrounding monolayer were between 90 and 110 ms, excitation emanating from micro-reentry alternately encountered entirely refractory or entirely excitable cells and therefore produced organized 2:1 conduction (Figure 4A and Supplementary Video 1). Here, quiescence was reached immediately following elimination of the micro-reentry (Supplementary Video 2). When mean APD in the cells of the surrounding monolayer were less than 90 ms or greater than 110 ms, sequential excitation from the micro-reentry encountered gradients in the ability of surrounding cells to be excited, led to asymmetric conduction block, and eventually produced chaotic activation. The duration of residual fibrillation after elimination of micro-reentry increased with decreasing APD (Supplementary Video 2). This increase was well-predicted by the fibrillogenicity index (Figure 5A).

We also examined the impact that the presence of micro-reentry had on the duration of fibrillation (experiment 2, Table 1 and Figure 5B). There was no difference between the duration of fibrillation episodes in monolayers without the substrate for structural micro-reentry and monolayers in which micro-reentrant substrate had been focally ablated (Figure 1C). In monolayers with a structural micro-reentrant substrate, the duration of fibrillation was increased (relative to monolayers without micro-reentrant substrate) only when the maximum possible excitation frequency of the surrounding tissue was lower than the excitation frequency of the structural micro-reentry.

### The Impact of Multi-Wavelet Reentry on Structural Mirco-Reentry

**FIGURE 6:**
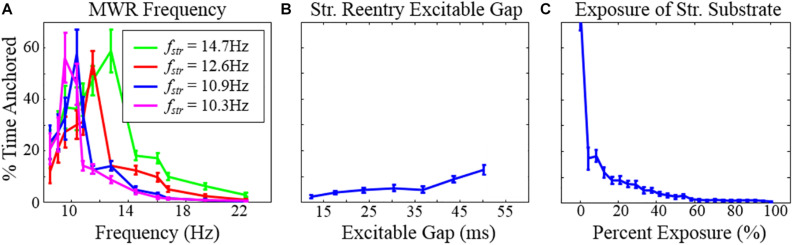
The percentage of time during which structural reentry was present around the circuit as a function of **(A)** the frequency of MWR in the tissue surrounding the micro-reentrant driver, **(B)** the excitable gap, and **(C)** the exposure of the micro-reentrant circuit to the surrounding tissue. Error bars show 95% confidence intervals. Note that **(A)** the percentage of time that there is reentry around the structural circuit is highest when the structural reentry and the frequency of fibrillation in the surrounding tissue is equal. When the micro-reentry is slower than the MWR frequency (right of peak), time anchored is reduced because (1) MWR provides a greater number of waves per unit time that can deanchor micro-reentry and (2) the greater frequency that MWR waves are produced favors collision of waves progressively closer to the slower micro-reentry circuit. Counterintuitively, when the micro-reentry is faster (left of peak), time anchored is reduced, in this case the longer wavelength of the surrounding tissue results in wave collision (micro-reentry vs. MWR), wave break and rotation immediately adjacent to the structural circuit. These waves then need only travel a short distance to interact with waves in the structural circuit (*f*_str_ = the frequency of micro-reentry around the structural micro-reentrant circuit). *This figure relates to experiments 3–5.

We next examined the dynamics of structural micro-reentry in the presence of surrounding MWR. The percentage of time that reentry was present around the scar substrate (time anchored) depended upon the relative frequencies of the micro-reentry and MWR (experiment 3, Table 1 and Figure 6A). When the frequency of the micro-reentry was slower than that of the MWR, the time anchored was reduced. When the frequency of the micro-reentry was faster than that of the MWR, time anchored was also reduced. Only when the frequency of the micro-reentry and MWR were equal was time anchored maximal. As the excitable gap of the structural reentrant circuit increased, so too did the probability of structural reentry (experiment 4, Table 1 and Figure 6B). Increasing exposure of the structural circuit to the surrounding MWR led to decreasing probability of structural reentry (experiment 5, Table 1 and Figure 6C).

### The Probability of Structural Macro-Reentry After Termination of Multiwavelet Reentry

**FIGURE 7:**
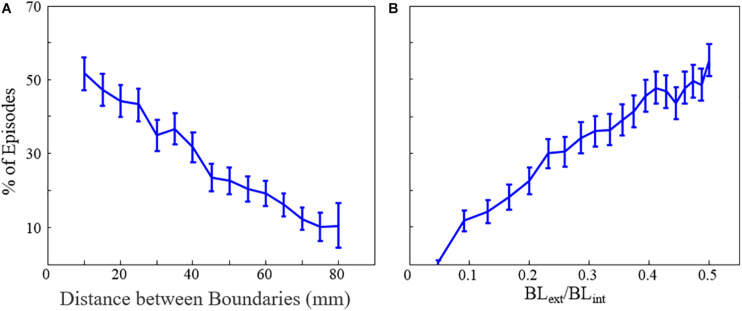
The percentage of fibrillation episodes that end in macro-reentrant tachycardia (rather than quiescence) as a function of **(A)** the distance between boundaries and **(B)** the ratio of internal and external unexcitable boundary lengths. Error bars show 95% confidence intervals. BL_ext_ = external boundary length, BL_int_ = internal boundary length. *This figure relates to experiments 6 and 7.

Finally, we explored the likelihood that MWR would convert to atrial tachycardia around a focal ablation scar (acting as the substrate for structural macro-reentry). We found that the probability of MWR converting to “atrial” tachycardia decreased linearly with increasing distance (experiment 6, Table 1 and Figure 7A) and disparity in size (experiment 7, Table 1 and Figure 7B) between the internal (focal ablation) and external (tissue edge) boundaries.

## Discussion

Multi-wavelet reentry and focal drivers have long been considered distinct, mutually exclusive mechanisms of fibrillation. It is often assumed that the chaotic activation surrounding a focal driver is a passive process whose perpetuation depends upon the presence of the focal driver. However, in both FD and MWR, the chaotic activation patterns are produced by wave break and shifting refractoriness. We propose here that the distinction between passive fibrillatory conduction and MWR could be more logically defined from a functional perspective: if wave break produces rotation that completes a full circuit it is reentry and can self-sustain (MWR), if circuits are not completed, activation is passive and driven, requiring a driver. As we have previously explored (Carrick et al., 2015), MWR falls along a continuum, and where a given piece of cardiac tissue will fall on this spectrum is defined by its size and degree of electrical derangement. In atria with relatively low propensity to fibrillate, changing propagation is passive and depends upon the presence of active drivers. With more extreme electrical derangement, variable conduction is self-sustaining, and may perpetuate even in the absence of a driver.

In the present study, we have explored a number of different aspects of the relationship between stationary drivers (in this case structural micro-reentry) and moving drivers (MWR) when they coexist during fibrillation. This relationship has traditionally been viewed as one sided; focal drivers act upon the surrounding excitable tissue to generate shifting conduction block and varying activation patterns (Ryu et al., 2005; Krummen et al., 2009). We found that these chaotic, randomly propagating waves can, through chance, be directed back toward the driver site that spawned them and interact with their own source. If the focal driver is reentrant (e.g., structural micro-reentry), that driver may be terminated by an appropriately timed collision with one of these external waves. Subsequently, again through chance, the chaotic waves can cause unidirectional block adjacent to a substrate for micro-reentry, reinitating the focal driver. Thus, the waves of fibrillation may both initiate and terminate focal reentrant drivers.

### Interactions Between Functional and Structural Reentry

**FIGURE 8:**
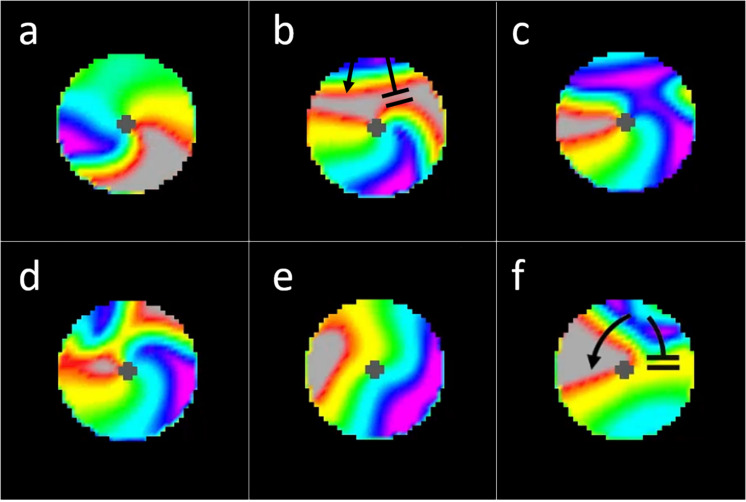
Interactions between incoming waves from chaotic activation surrounding a micro-reentrant circuit and waves within the micro-reentrant circuit. In these images we see only the tissue within the structural micro-reentrant circuit, the surrounding tissue is masked for clarity. The opening in the scar that protects this circuit is at the top of the circuits displayed (from 10:00 to 2:00 o’clock). Top row **(a)** an anchored reentrant wave propagating counter-clockwise around the central scar, **(b)** an incoming external wave is seen entering the top of the circuit, this wave collides with the reentrant wave front (top right) but does not collide with the receding reentrant wave tail, **(c)** the result is resetting of the micro-reentry. **(d)** here an incoming external wave collides with both the reentrant wave front and the reentrant wave tail, causing bidirectional block and **(e)** termination/deanchoring of micro-reentry. **(f)** Finally we see a subsequent incoming wave, the portion of the incoming wave that travels clockwise around the structural circuit collides with (and blocks) against the receding tail of a prior (non-reentrant) wave that had entered the circuit. Meanwhile, the counter-clockwise portion of the incoming wave does not collide with the prior waves receding tail. The result is unidirectional block and re-initiation of micro-reentry around the structural circuit [colors represent membrane voltage (purple—maximum voltage, red—threshold voltage, gray—sub-threshold voltage). *This figure relates to experiments 3–5.

In the presence of both a scar substrate for reentry and surrounding cardiac tissue with sufficient propensity to fibrillate, structural and moving functional reentry coexisted and together drove fibrillation (Lee et al., 2014). We found that these patterns of activation did not exist independently from one another; there was consistent interaction between the two. Micro-reentrant drivers produced and perpetuated moving functional reentry, which in turn terminated (Figures 8a–c and Supplementary Video 3) and then reinitiated that same driver (Figures 8d–f). The relative probabilities of these different events depended upon the properties of the structural reentrant substrate and the tissue that surrounded it. Both initiation and termination of structural micro-reentry required access of waves to the central scar. As the APD of the surrounding cardiac tissue decreased, the frequency of the fibrillation it supported increased. Thus, the number of waves, and therefore the number of interactions with the substrate for micro-reentry, increased as well. This led to a corresponding decrease in the percentage of time that there was stable reentry around the reentrant substrate; the circuit was under constant bombardment by incoming waves. Likewise, increasing the exposure of the obstacle to the surrounding cardiac tissue allowed waves to approach the structural circuit from more directions, and therefore also caused a decrease in the likelihood of stable wave anchoring. When the frequency of fibrillation and structural micro-reentry were the same, the percentage of time during which waves were anchored to the obstacle peaked. Neither the stationary driver nor the surrounding fibrillation was able to consistently influence the other, and once initiated, micro-reentry was able to continue largely unmolested. Counter-intuitively, when the surrounding cardiac tissue has long APD (low frequency) this also caused a drop in the likelihood of stable micro-reentry. In this case, waves emitted by the relatively fast reentrant circuit collided with heterogeneously refractory cells in the directly adjacent cardiac tissue. This led to wave break in the immediate vicinity of the micro-rentrant circuit, producing waves well-positioned to reenter the circuit path and interrupt reentry. Finally, increasing the excitable gap led to an increase in the probability of stable micro-reentry. More space between an anchored wave’s front and refractory tail made it more robust to perturbations from external waves.

### Atrial Tachycardia Around Focal Obstacles Following Termination of Fibrillation

**FIGURE 9:**
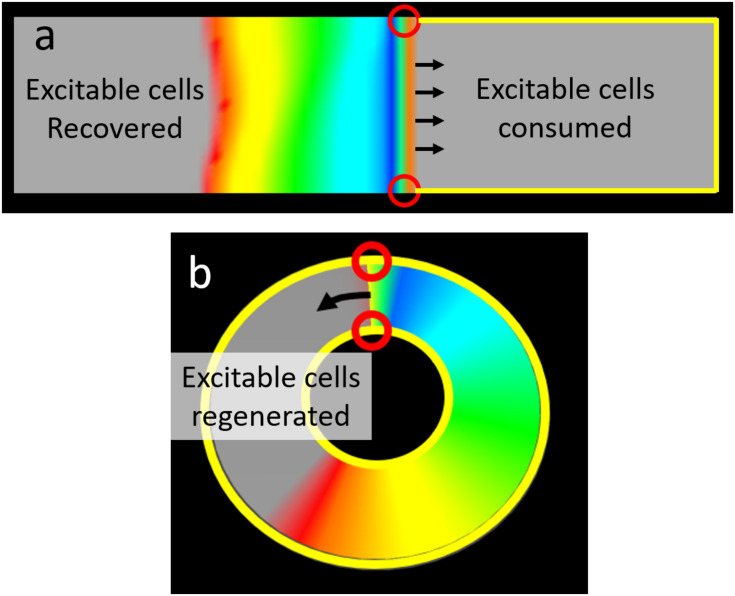
The relationship between wave ends and boundaries determines the capacity to support reentry. **(a)** In an uninterrupted rectangular monolayer of myocytes a planar wave front has two ends each on the outer boundary of the tissue. There is only one boundary on such a tissue, thus both wave ends are on the same boundary. In this configuration there is no continuity between the region (right side of figure) in front of the propagating wave (where excitable cells are consumed) and the region (left side of figure) where repolarization “regenerates” excitable cells. Thus activation will inevitably exhaust all the excitable cells in front of the wave and propagation will cease (no reentry). **(b)** This ring shaped tissue has both an outer boundary and a separate inner boundary. Here the wave has one end on each boundary. As a result there is an uninterrupted path between the wave front (where excitable cells are consumed and the wave tail (where excitable cells are regenerated). The result is a wave that will perpetually encounter excitable cells and hence reentry can perpetuate indefinitely [Colors represent membrane voltage (purple—maximum voltage, red—threshold voltage, gray—sub-threshold voltage].

One of the well-known complications associated with focal ablation of atrial fibrillation is the induction of atrial tachycardia (Wong et al., 2015; Teunissen et al., 2016; Yang et al., 2016). However, while focal ablation provides a substrate for macroscopic structural circuits to form, structural reentry does not occur in every instance. To understand why and when atrial tachycardia is likely to be induced, it is helpful to consider fibrillation as a randomly fluctuating population of waves. Each of these waves has an excitation front, a refractory tail, and two wave ends. Wave ends may be either free-floating, in which case they may form a functional reentrant circuit, or be anchored to boundaries. From the probabilistic viewpoint, functional reentry continues until all free-floating wave ends are anchored to unexcitable boundaries. In the case of uninterrupted sheets of cardiac tissue, there is only one boundary for wave ends to anchor to Figure 9a and Supplementary Video 3. The wave front spanning those two wave ends is presented with a diminishing supply of excitable cells and will extinguish against the boundary when its supply is exhausted. In cardiac tissue with a substrate for structural reentry (e.g., vein orifice or focal ablation), waves may anchor to two separate boundaries: the tissue edge and the structural obstacle. If the two wave ends are on different boundaries (e.g., the external boundary of a piece of cardiac tissue and an ablation lesion), the wave front encounters a replenishing supply of excitable cells and will propagate indefinitely as macro-reentrant atrial tachycardia (Figure 9b). In its most abstracted form, a circuit is simply that tissue/wave configuration in which there is uninterrupted access of the wave front of excitation (where excitable cells are “consumed”) to the wave tail of recovery (where excitable cells are regenerated).

Thus, properties which affect the likelihood of wave ends anchoring to separate boundaries affect the likelihood of atrial tachycardia. Boundaries that are further apart from one another or are more disproportionate in size reduce the probability of a single wave having wave ends anchored on opposite boundaries.

### Limitations

Our goal in this series of experiments was to establish the theoretical principles that define interactions between stationary and moving drivers of fibrillation. In particular, we were interested in defining qualitative relationships between emergent behaviors (e.g., structural reentry, fibrillation) and tissue properties (e.g., cellular APD, structural obstacles with varying degrees of protection). The computer model of propagation we selected for this study is therefore ideal, since its low computational burden allows for both direct visualization of electrical activity and exhaustive search of the parameter landscape. As with all *in silico* studies however, we cannot consider the concepts we present here to be confirmed. Our conclusions require validation in a biological setting, ultimately humans. Nonetheless, our findings provide a set of testable predictions that can guide the design of future experimental study.

Several recent modeling studies (Ali et al., 2019; Roney et al., 2020; Roy et al., 2020) have highlighted the importance of fibrosis on the results of AF ablation. While we employed a crude scar as a substrate for structural reentry, these studies employed extremely detailed anatomy using LGE MRI to identify regions of fibrosis. They demonstrated (1) that regions of fibrosis can form structural circuits and anchor reentrant drivers, (2) that ablation lesions themselves can form the substrate for reentry, and (3) that not all substrates for structural reentry will harbor a reentrant driver at the time of mapping, but can be the source of recurrence following ablation. These studies speak to the importance that structural circuits play in maintenance of AF. However, due to the computational burden of the models used, a very limited number of simulations could be performed and those simulations were brief (e.g., 15 s). They were therefore not able to characterize the rate of structural reentry, the dynamics of anchoring/deanchoring, or interactions with chaotic activation. Our experiments, when taken in the context of this work from more detailed models, offer a window into the complex, dynamic interactions between stationary and moving drivers of AF.

There is an enormous amount of complexity involved in human atrial fibrillation that was not captured by the simplistic model we used in this study. Our intention was not to determine what does happen in human AF but rather what can happen when a generic stationary driver is surrounded by tissue capable of supporting moving functional reentrant circuits. The use of a simplistic model allowed us to test long episodes of fibrillation, in tissues with a wide range of parameters and with multiple episodes of fibrillation in each tissue. Our results therefore speak more to how focal drivers and fibrillation can interact and how some physiologic features affect those interactions; the exact numerical values of our results cannot be directly extrapolated to human AF. The actual behavior in any individual AF patient will be determined by the extremely complex interactions between that patient’s electrical properties and tissue architecture; our data provides a framework for understanding the complex propagation that emerges.

## Conclusion

In this study, we used a computational model to examine the interplay between spatially stable structural micro-reentry and the surrounding cardiac tissue during fibrillation. Focal drivers could initiate fibrillation via wave break in the setting of heterogeneous refractoriness of the surrounding cardiac tissue, and chaotic activation could both initiate and terminate structural micro-reentry via well-timed collisions between waves of excitation. The degree to which a focal driver and the surrounding chaotic activation interact depended on the relative characteristics of the structural and functional reentrant substrates. In order to accurately describe cardiac tissue’s propensity to support fibrillation, the relationship between stationary and moving drivers must be taken into account.

## Data Availability Statement

The raw data supporting the conclusions of this article will be made available by the authors, without undue reservation, to any qualified researcher.

## Author Contributions

PS: study design, data analysis, and writing. RC: study design, data acquisition, data analysis, and writing. BB and OB: data analysis. All authors contributed to the article and approved the submitted version.

## Conflict of Interest

PS received consulting fees from Biosense Webster and Medtronic. The remaining authors declare that the research was conducted in the absence of any commercial or financial relationships that could be construed as a potential conflict of interest.

## Footnotes

1 www.uvm.edu/∼vacc/
